# Supplementary material for: Chlorine Dioxide Degradation Issues on Metal and Plastic Water Pipes Tested in Parallel in a Semi-Closed System
Source: Int J Environ Res Public Health. 2019 Nov 19;16(22):4582. doi: 10.3390/ijerph16224582 (PMC6888174; doi:10.3390/ijerph16224582)
Supplement: Supplementary file 1 [file ijerph-16-04582-s001.pdf]

Chlorine dioxide degradation issues on metal and plastic water pipes tested in parallel in a semi-closed system

Alberto Vertova[1], Giordano Lesma[1,2], Sandra Rondinini[1], Alessandro Minguzzi[1], Luigi Falciola[1,4], Alessandro Miani[3,4], Marco Aldo Ortenzi,[1,2*]

1 Department of Chemistry, Università degli Studi di Milano, Via Golgi 19 – 20133 Milan
2 CRC Materiali Polimerici (LaMPo), Department of Chemistry, Università degli Studi di Milano, Via Golgi 19 - 20133 Milan, Italy
3 Department of Environmental Science and Policy (ESP), Università degli Studi di Milano, Via Celoria 2 – 20133 Milan
4 Italian Society of Environmental Medicine (SIMA), Via Monte Leone 2 – 20149 Milan
* Correspondence: marco.ortenzi@unimi.it; Tel.: +39-0250314135 (F.L.)

Supporting Information

Electrochemical Impedance Measurements

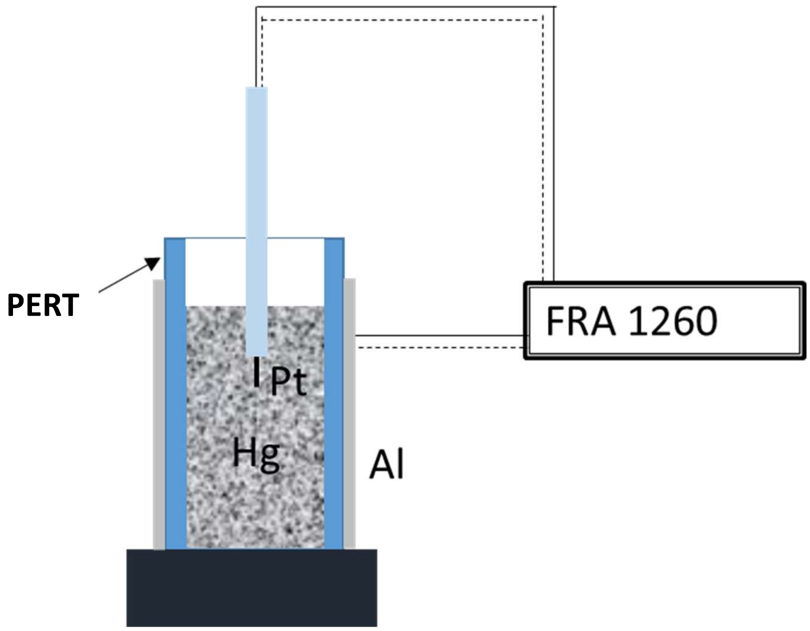

Figure S1: Experimental setup for 4 wires Impedance Measurements on PERT with Al shield.

EDS Analyses

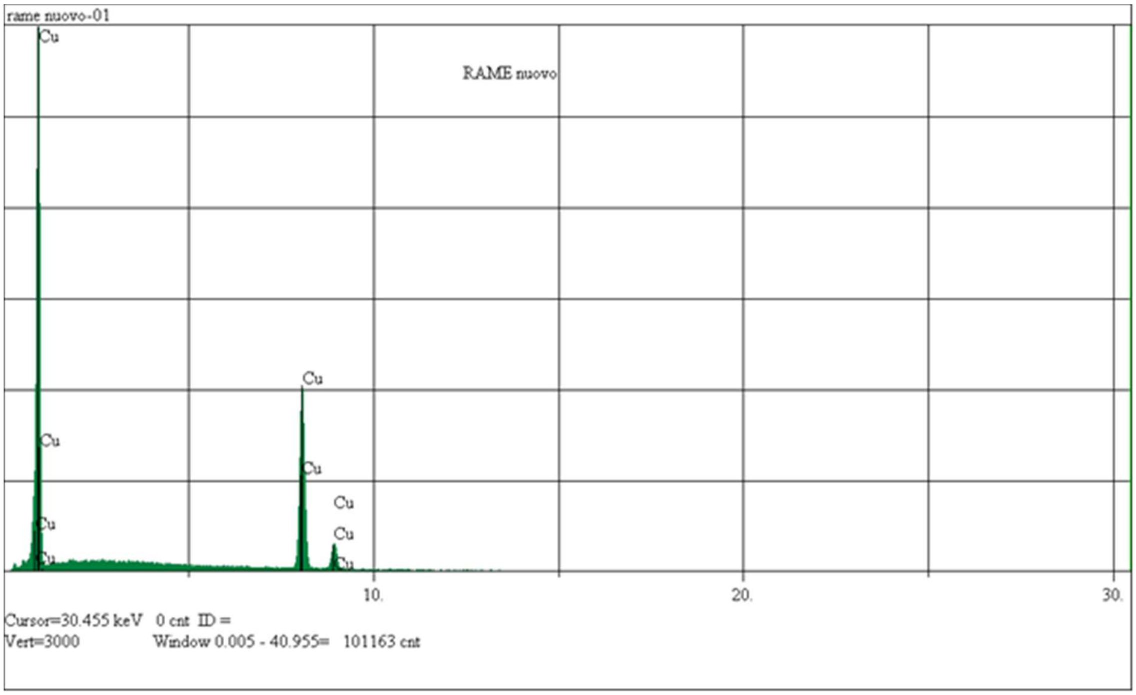

Figure S2a: Copper – New pipe

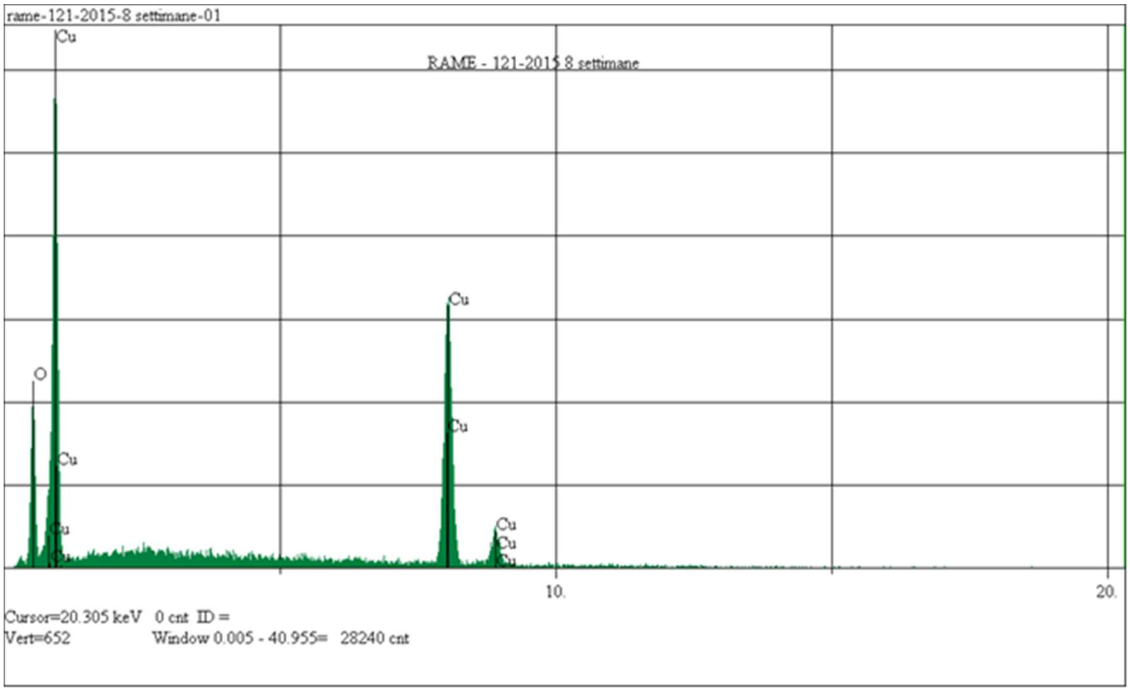

Figure S2b: Copper pipe Aged 8 weeks

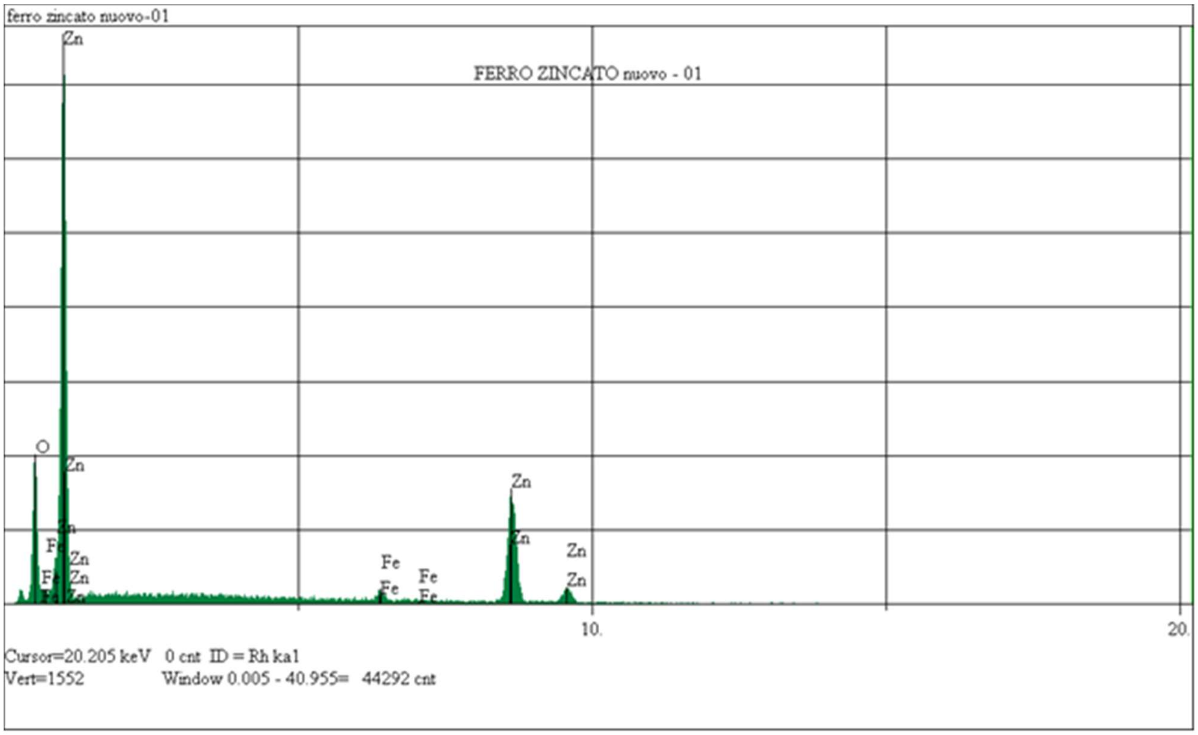

Figure S3a: Galvanized Steel – New pipe

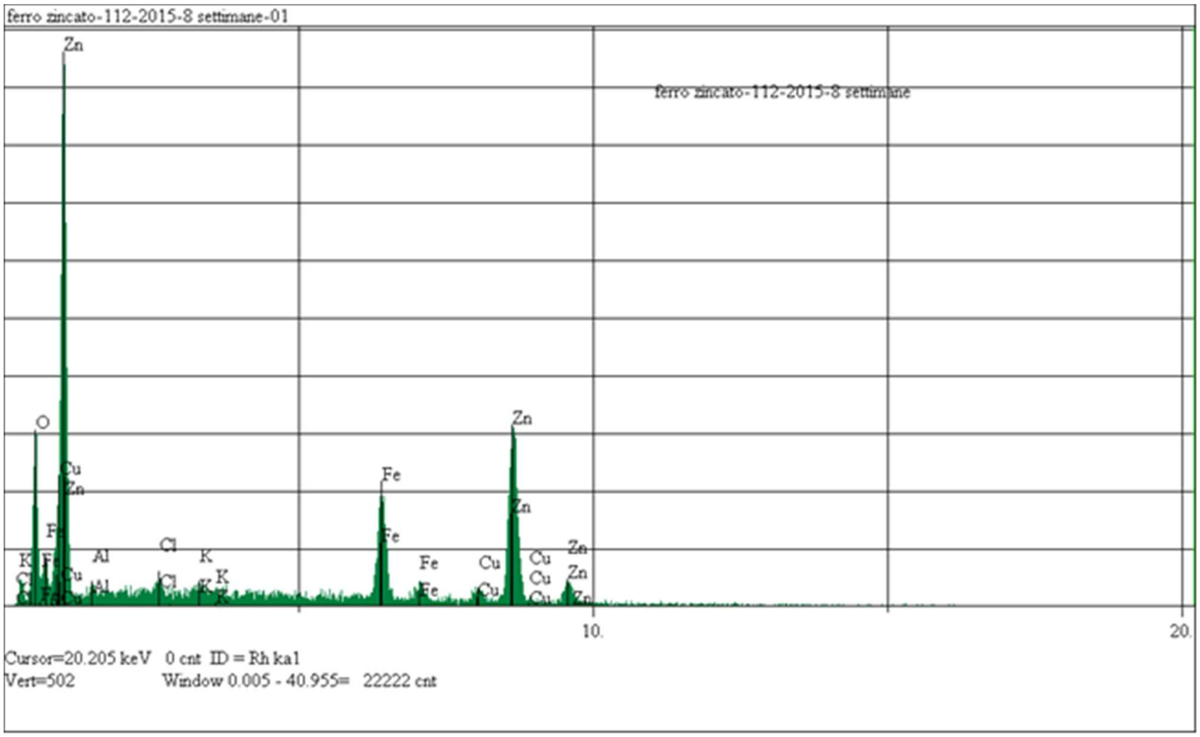

Figure S3b: Galvanized Steel pipe aged 8 weeks

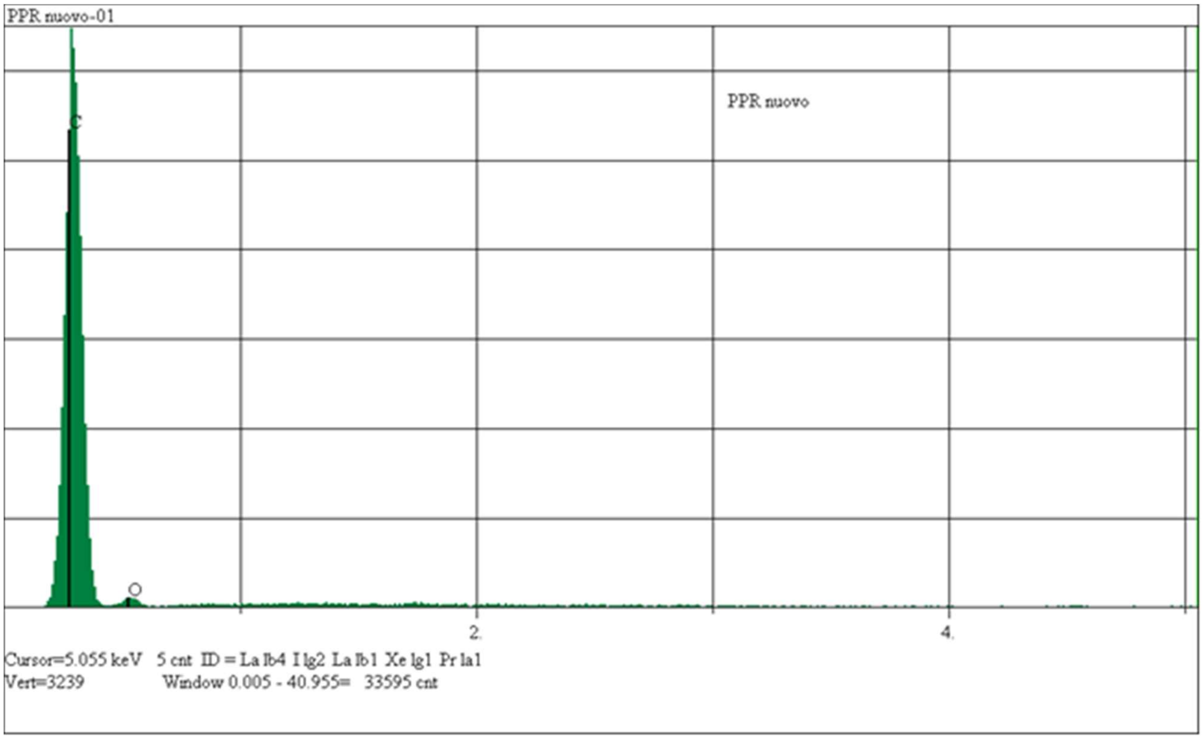

Figure 4Sa: PPR – New pipe

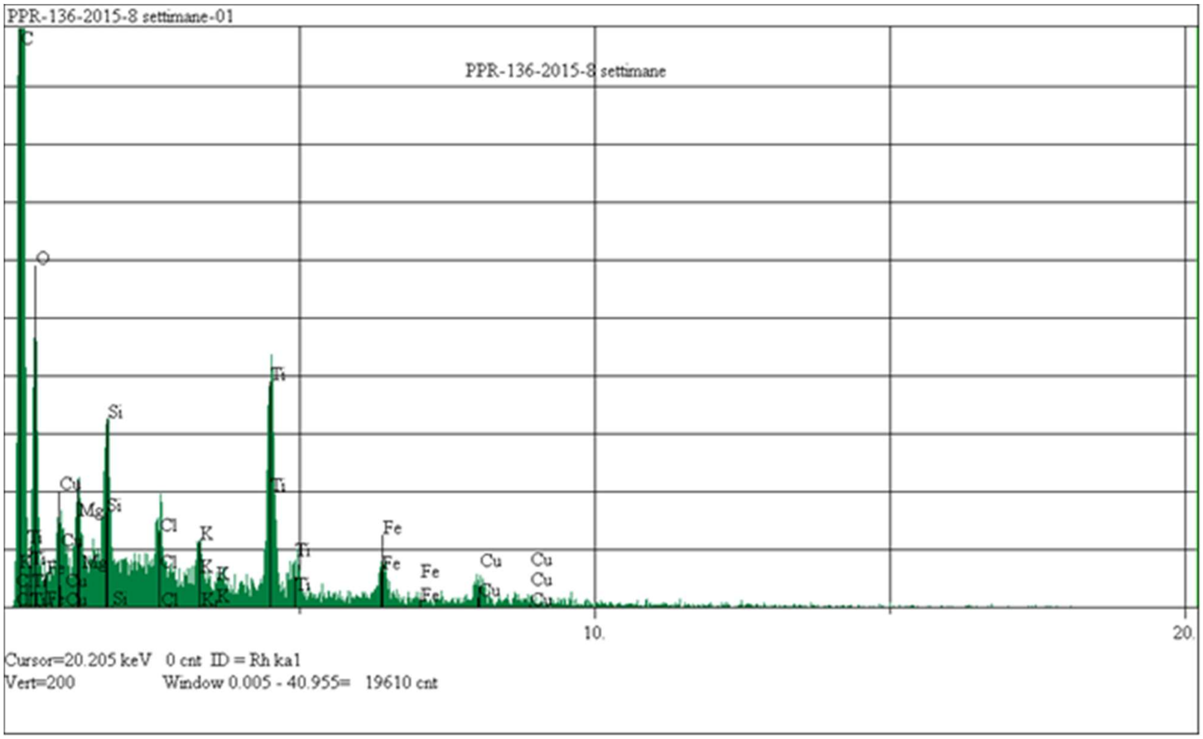

Figure 4Sb: PPR pipe aged 8 weeks

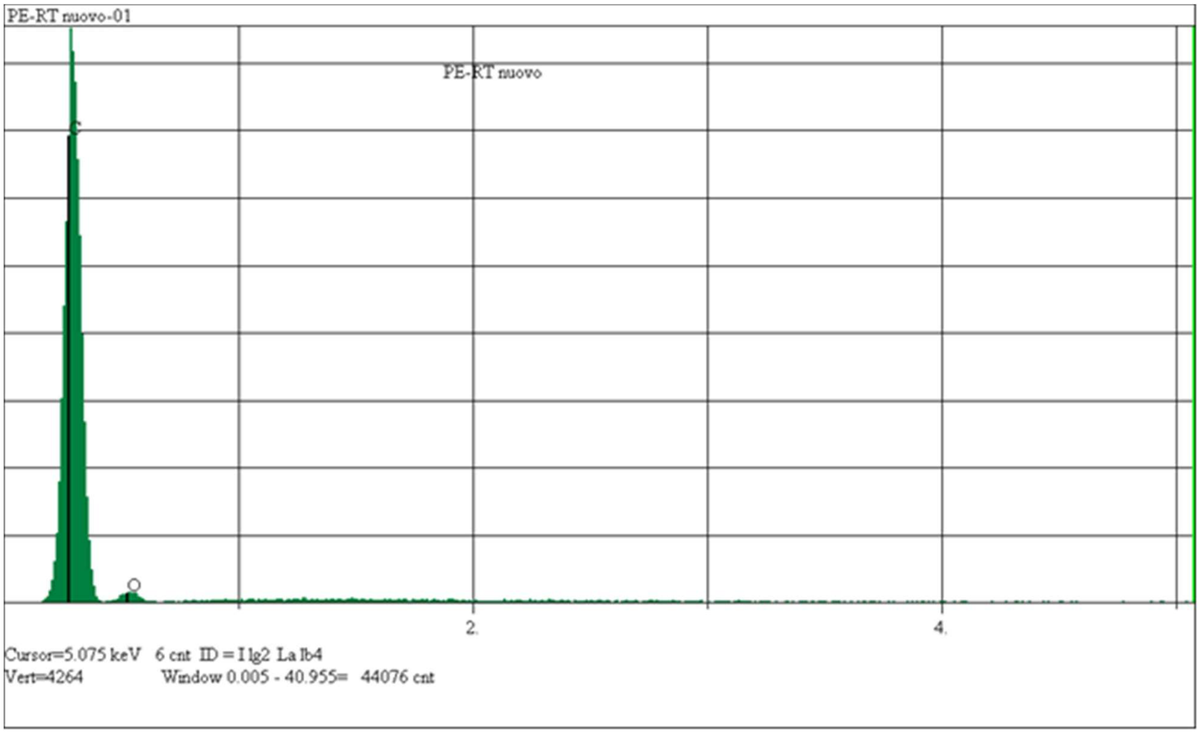

Figure 5Sa: PERT – New pipe (multilayer)

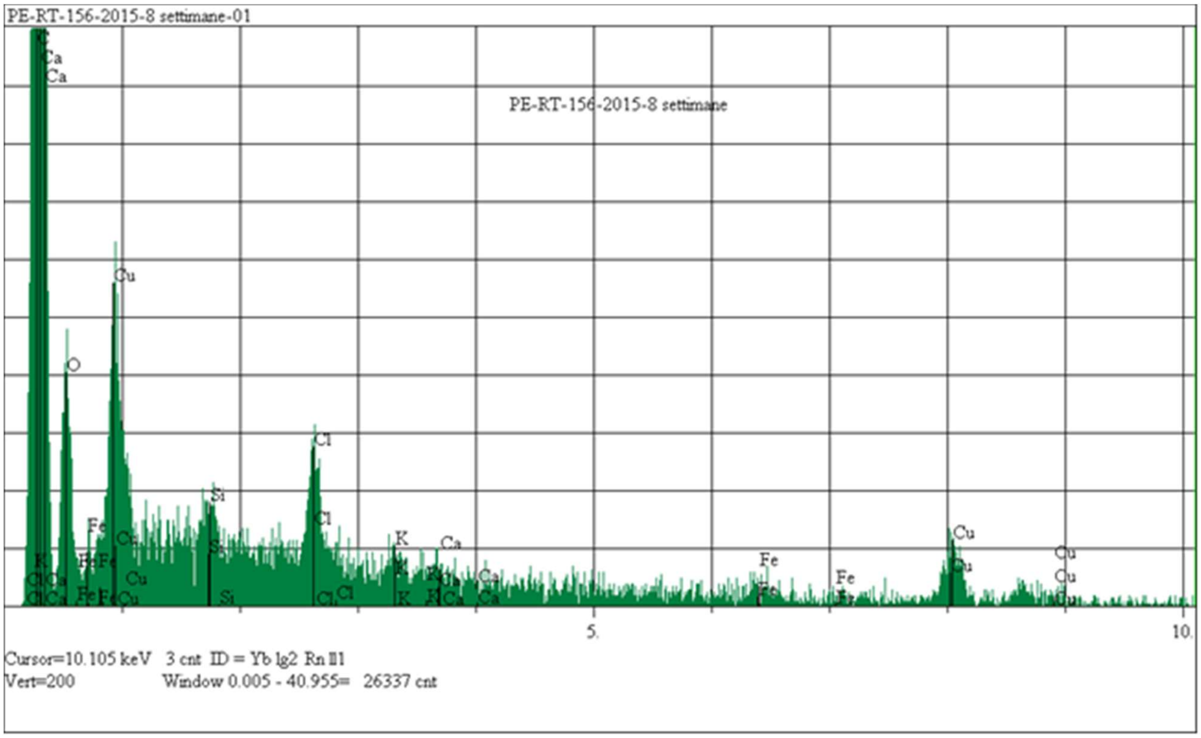

Figure 5Sb: PERT multilayer pipe aged 8 weeks

FT-IR analyses (bulk)

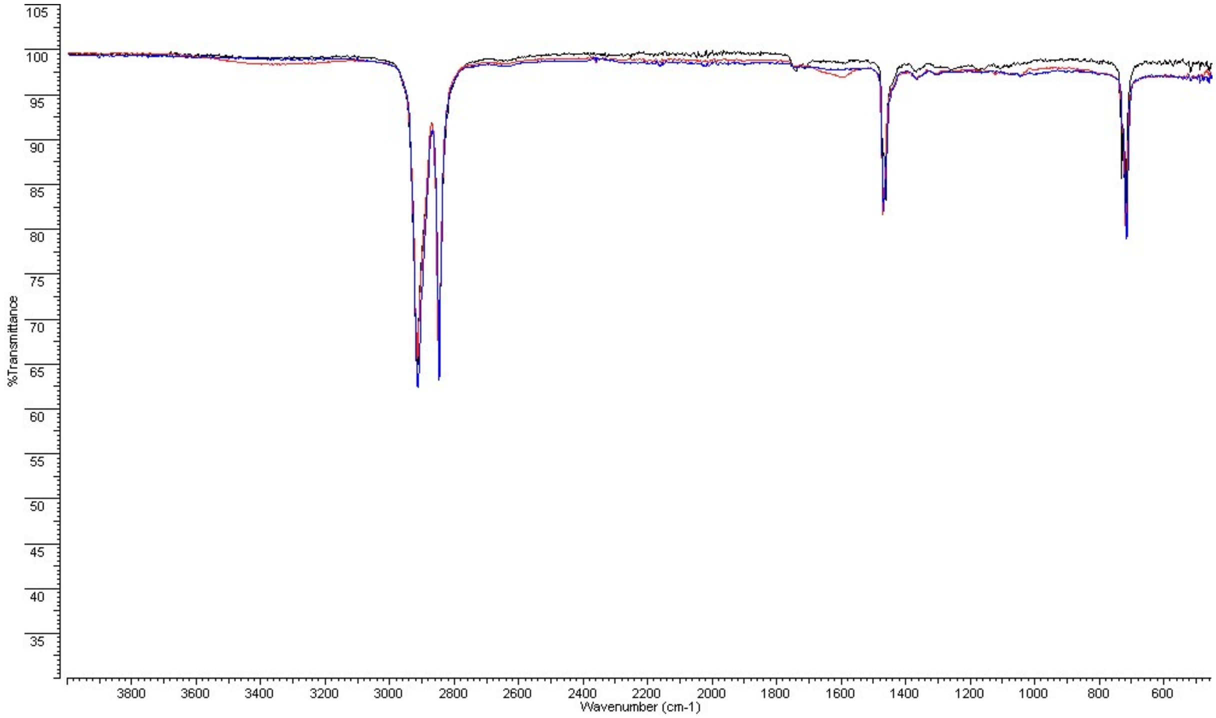

Figure S6: FT-IR of PERT bulk: new (black), 4 weeks (red), 8 weeks (blue)

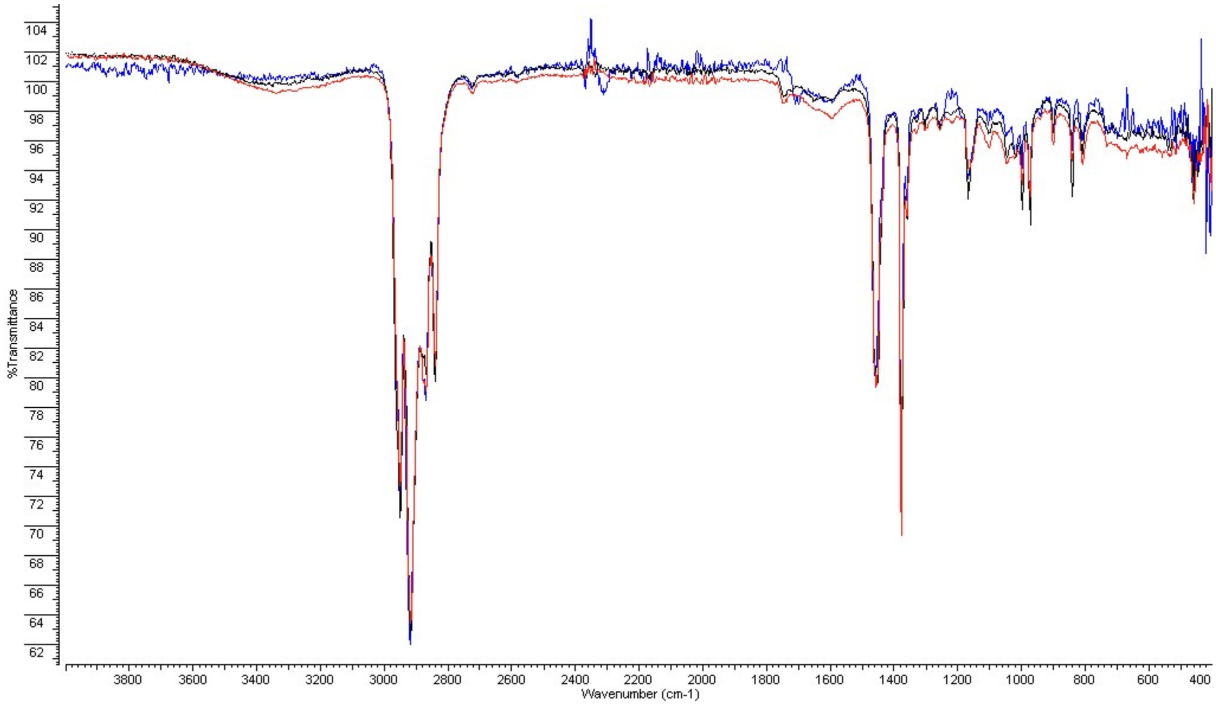

Figure S7: FT-IR of PPR bulk: new (black), 4 weeks (red), 8 weeks (blue)

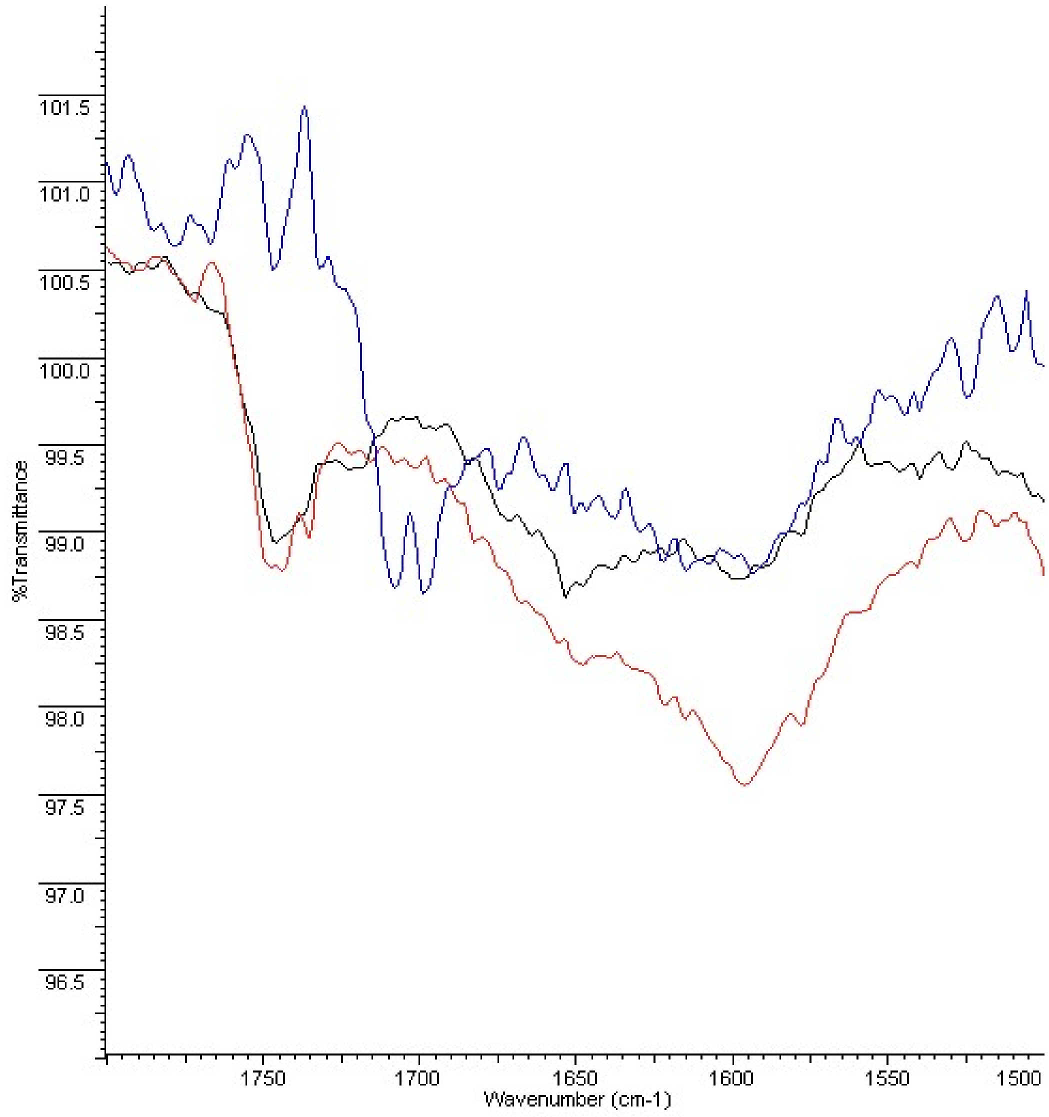

Figure S8: FT-IR spectra of PPR bulk: new (black), 4 weeks (red), 8 weeks (blue) – Magnification of 1800-1500 cm^{-1} region

DSC Analyses

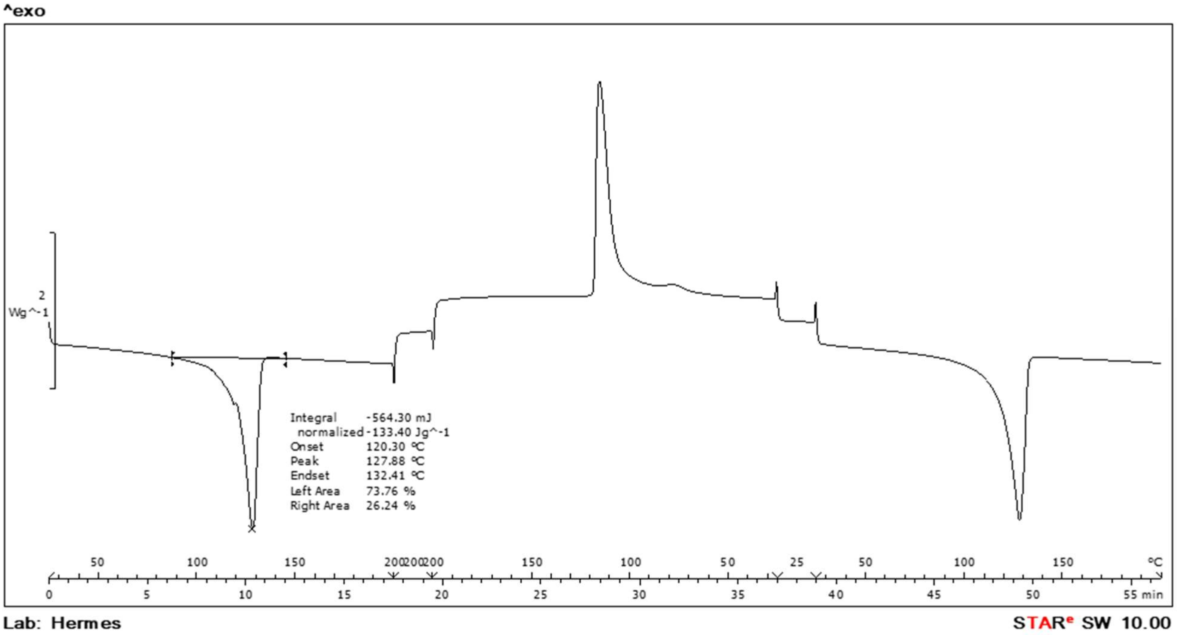

Figure S9: DSC analyses (First heating – cooling – Second heating) on PERT new pipe

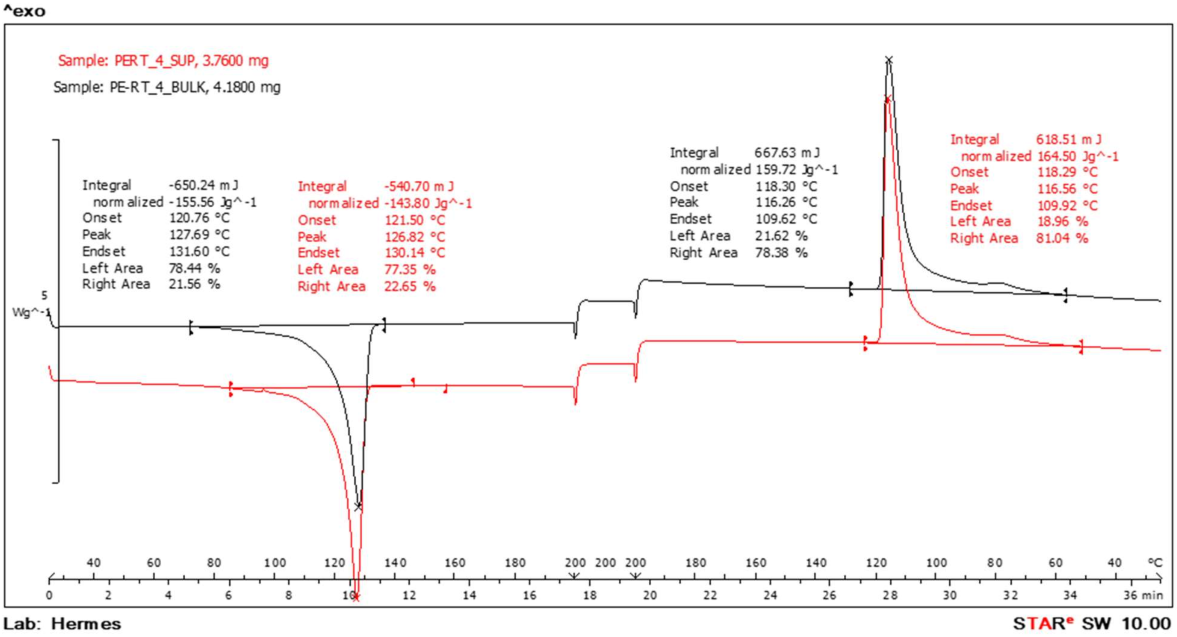

Figure S10: DSC analyses (First heating – cooling) on PERT pipe aged 4 weeks: bulk (black), surface (red)

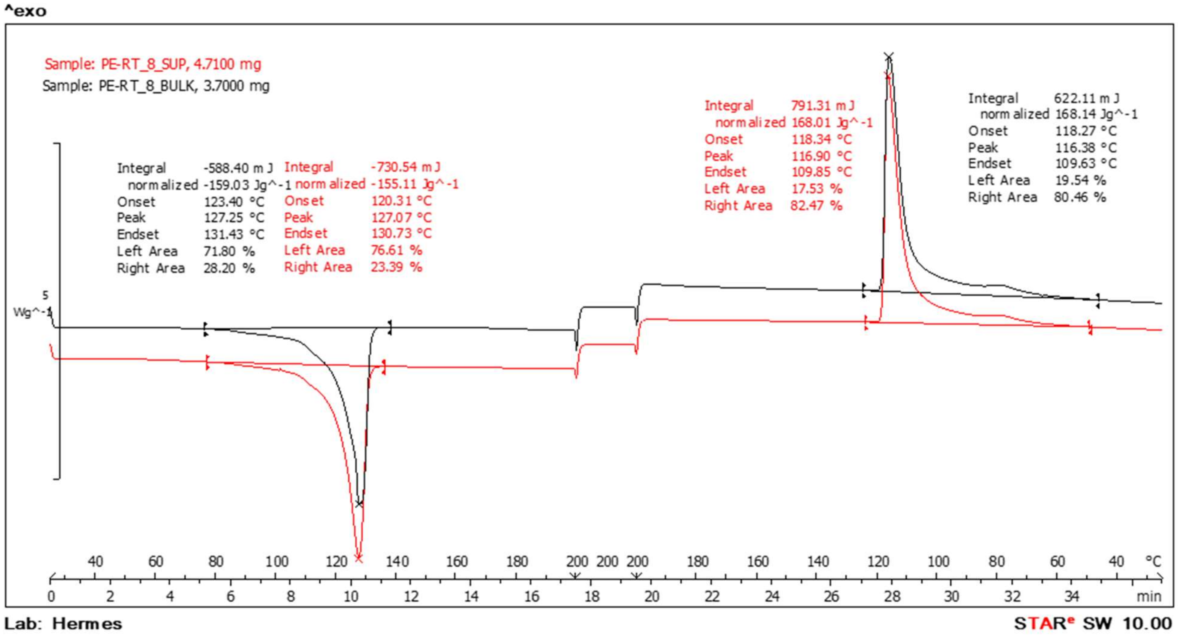

Figure S11: DSC analyses (First heating – cooling) on PERT pipe aged 8 weeks: bulk (black), surface (red)

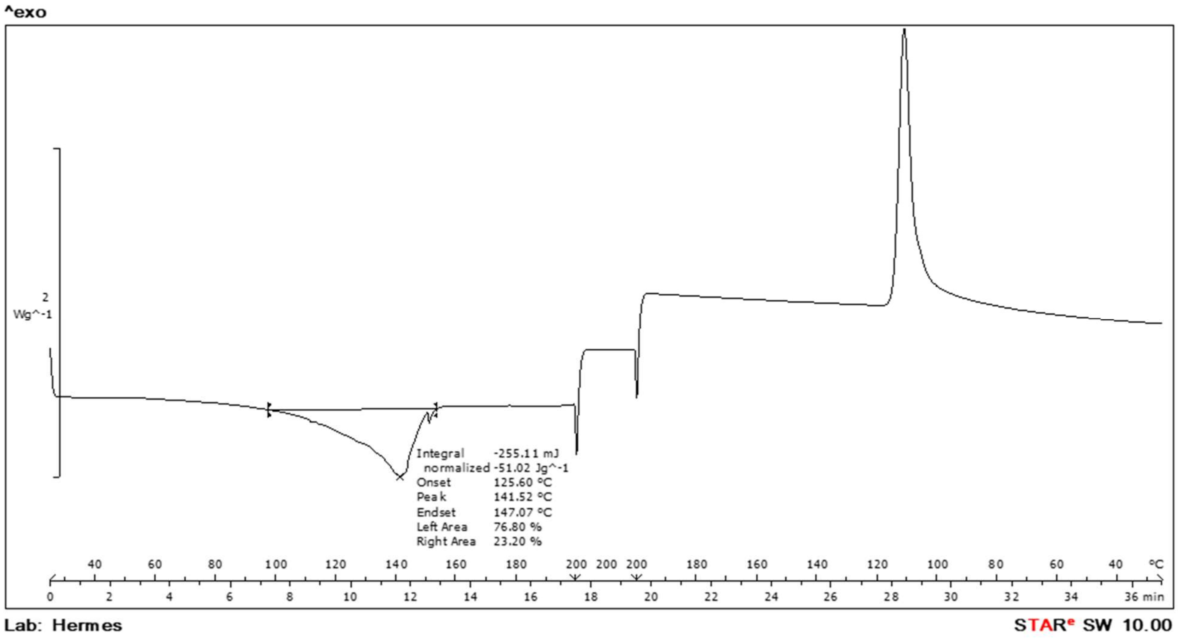

Figure S12: DSC analyses (First heating – cooling) on PPR new pipe

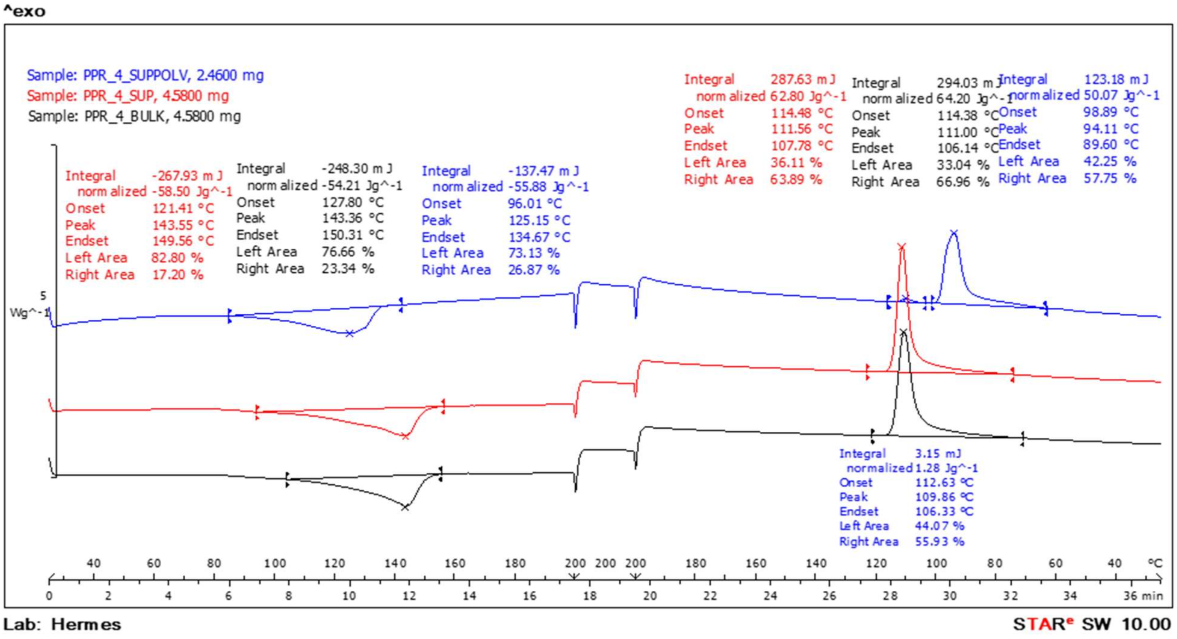

Figure S13: DSC analyses (First heating – cooling) on PPR pipe aged 4 weeks: bulk (black), surface (red), surface powder (blue)

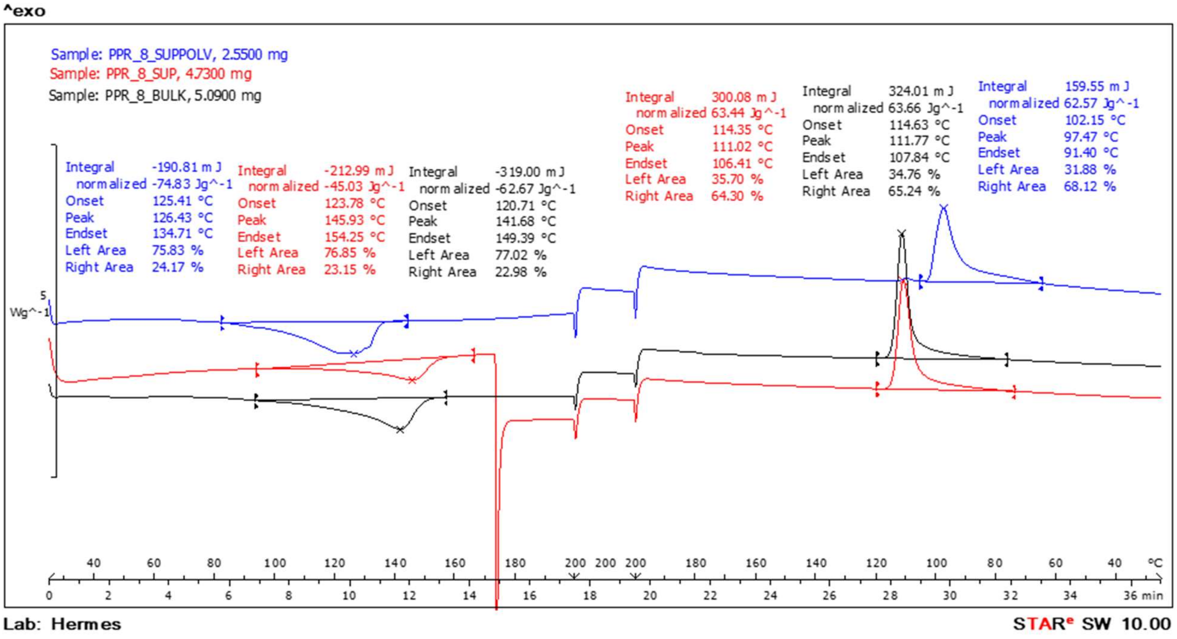

Figure S14: DSC analyses (First heating – cooling) on PPR pipe aged 8 weeks: bulk (black), surface (red), surface powder (blue)
